# Characterization of the Immunoglobulin Domain of *C. elegans* SISS-1/EGF

**DOI:** 10.17912/micropub.biology.002058

**Published:** 2026-03-11

**Authors:** Jesse G Jones, Cheryl Van Buskirk

**Affiliations:** 1 Biology, California State University, Northridge, Northridge, CA, US

## Abstract

Immunoglobulin-like domains (IgDs) are widespread among cell surface proteins and participate in cell adhesion and signaling. A subset of Epidermal Growth Factor (EGF) family ligands possess an IgD, with variable impact on EGF signaling. *C. elegans* SISS-1 is an Ig-EGF released by damaged cells that promotes sleep, and its IgD is necessary for this endogenous function. Here we use SISS-1 ectopic expression to further investigate IgD function, and our data confirm the critical role of the SISS-1 IgD in sleep. We also examine the impact of adding the SISS-1 IgD to another *C. elegans* EGF, LIN-3 .

**Figure 1. The SISS-1 IgD is necessary for SISS-1/EGF signaling but has little impact when added to LIN-3/EGF:**
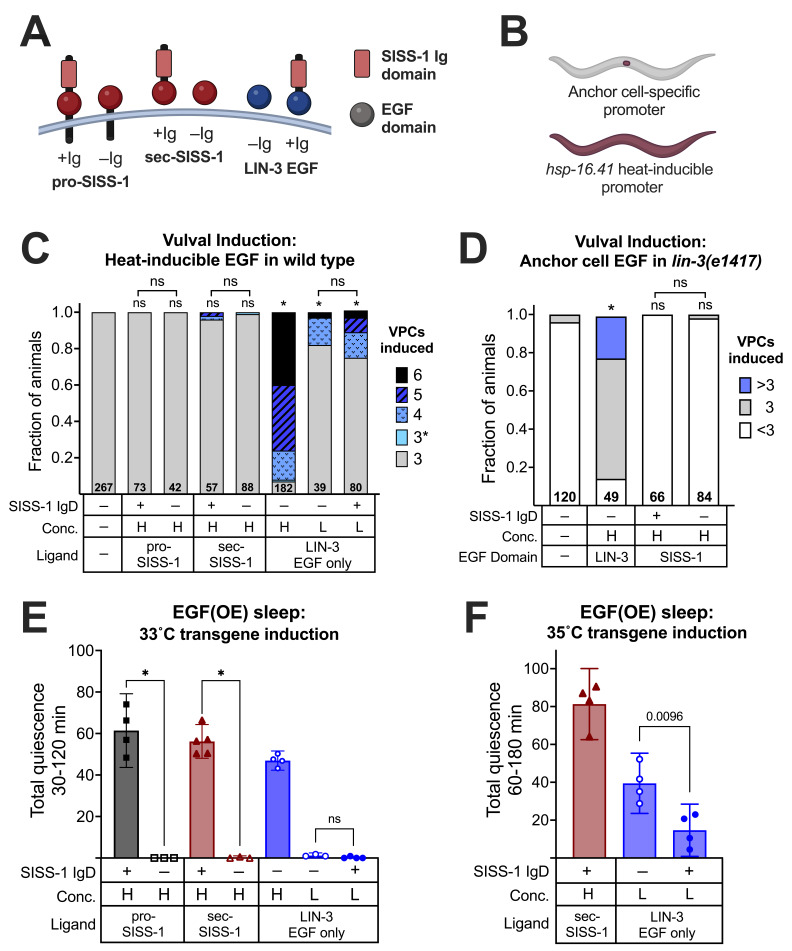
A) SISS-1 and LIN-3 protein variants examined in this study. Ig = immunoglobulin-like domain; EGF = Epidermal Growth Factor-like domain. The curved line represents the cell membrane. B) The two promoters used in this study, driving expression either specifically within the anchor cell or ubiquitously following heat shock, allowing examination of EGFR activation in multiple contexts. (C,D) Vulval precursor cell (VPC) induction inferred by vulval morphology at the L4 stage, with the number of animals examined at the base of each bar. The presence of the SISS-1 IgD is indicated (+/–), and H and L refer to high and low transgene concentrations, described in methods. ns = not significant (P>0.05), *P<0.0001 Fisher's exact test with Holm correction vs. non-transgenic animals or as indicated by brackets. C) VPC induction following EGF overexpression by a 33˚C heat shock at the late L2 stage in a wild-type background. In wild-type animals, three VPCs are induced. In EGF(OE) animals, up to six VPCs adopt vulval fates.&nbsp; D) VPC induction in *lin-3 ( e1417 )* animals carrying an EGF transgene under the control of an anchor cell-specific promoter. In *lin-3 ( e1417 )* animals, fewer than three VPCs are typically induced. (E,F) Behavioral quiescence (cessation of movement and feeding) following widespread EGF overexpression at the young adult stage, with transgene induction either at 33˚C (E) or at 35˚C (F). The heat shock also triggers endogenous stress-induced sleep (SIS) (Hill et al. 2014), and thus transgene-dependent sleep is measured from the end of SIS, corresponding to 30 min (33˚C) or 60 min (35˚C) after heat shock. Behavior was scored every 15-30 min, and total quiescence is presented here as the mean area under the curve of the time courses, with 95% confidence intervals. Each data point represents one time course of roughly 25 animals. For panel E, ns = not significant, *P<0.05, Kruskal-Wallis test ( LIN-3 EGF high excluded) with Dunn's multiple comparisons test. For panel F, the P value was derived from a two-tailed unpaired t test.

## Description

Ig-like domains (IgDs), first characterized within immunoglobulin proteins (Williams & Barclay 1988), are highly abundant among integral membrane proteins that act as cell adhesion molecules (CAMs) within the nervous system (Yoshihara et al. 1991). Neural Ig-CAMs participate in homophilic and heterophilic interactions, forming networks critical for nervous system development (Zinn & Özkan 2017). IgDs can also be found in a subset of Epidermal Growth Factor (EGF) family ligands, membrane proteins with an extracellular EGF-like domain that is shed from the cell surface to activate EGF receptors (EGFR) nearby or at a distance (Schneider & Wolf 2008). Within these Ig-EGFs, the IgD lies membrane-distal to the EGF domain (Uniprot Consortium 2025). The impact of the IgD on EGF signaling appears to vary. In the case of vertebrate Neuregulin 1, the IgD likely enhances receptor interaction (Li & Loeb 2001; Eto et al. 2006; Centa et al. 2018), though one study found the IgD to have an inhibitory effect on signaling (Warren et al. 2006). The IgD of *Drosophila* Vein appears to restrict ectopic EGF signaling (Donaldson et al. 2004).

Among *C. elegans* Ig-EGFs, the IgD appears to be critically required for ligand function. SISS-1 is an Ig-EGF that appears to be released by damaged tissues, promoting EGFR activation within sleep-promoting neurons (Hill et al. 2024). Loss of the *siss-1* IgD produces the same sleep defect as a *siss-1* gene deletion (Jones & Van Buskirk 2025). This strict IgD-dependence is observed with another *C. elegans* Ig-EGF, IGEG-2 (Mendez et al. 2025). The endogenous function of IGEG-2 is unknown, and the necessity of its IgD was assessed via IGEG-2 overexpression phenotypes, which include EGFR-dependent hypersomnia as well as vulva cell fate hyperinduction (Mailhot et al. 2025). Vulval induction is normally mediated by the non-IgD-containing EGF family ligand LIN-3 (Hill & Sternberg, 1992), which is released by the anchor cell and promotes vulval fates in the three nearest of six EGFR-expressing vulval precursor cells (VPCs) (Moghal & Sternberg 2003).

Tissue-general overexpression (OE) of any of the three *C. elegans* EGF family ligands promotes hypersomnia (Van Buskirk & Sternberg 2007; Hill et al. 2024; Mailhot et al. 2025), and LIN-3 (OE) and IGEG-2 (OE) can promote vulval hyperinduction, with up to six VPCs adopting vulval fates (Katz et al. 1995; Mailhot et al. 2025). Interestingly, overexpression of pro- SISS-1 has no impact on vulval induction, and a constitutively-secreted SISS-1 promotes only mild vulval hyperinduction, with 4 VPCs induced in a small fraction of animals (Hill et al. 2024). We wished to determine whether the general failure of SISS-1 (OE) to promote vulval induction is attributable to its IgD. For example, given the involvement of IgD-containing proteins in neural adhesion in other animals (Yoshihara et al. 1991), the SISS-1 IgD might concentrate the shed ligand at sleep-promoting neurons and away from peripheral tissues including the VPCs. This model also helps explain the sleep defect exhibited by *siss-1* mutants missing only the IgD (Jones & Van Buskirk 2025). Here we test this model by expressing EGF ligands with and without the SISS-1 IgD ( Fig. 1A ), in both tissue-specific and tissue-general contexts ( Fig. 1B ).

We reasoned that if the SISS-1 IgD serves to bias the ligand toward neuronal EGFR activation, deletion of the IgD should increase SISS-1 signaling within peripheral tissues, resulting in increased VPC induction. Conversely, adding the SISS-1 IgD to LIN-3 might reduce its vulval induction function. We first examined the impact of the IgD on vulval induction by widespread overexpression of full-length pro- SISS-1 and constitutively-secreted sec- SISS-1 . Contrary to our expectation, loss of the IgD did not enhance vulval induction by SISS-1 (OE) ( Fig. 1C ). We then examined the impact of the addition of the SISS-1 IgD to the isolated EGF domain of LIN-3 (sec- LIN-3 EGF), which causes vulval induction when overexpressed (Katz et al. 1995, Fig. 1C ). We were unable to isolate viable transgenic animals carrying LIN-3 with the SISS-1 IgD (sec- LIN-3 EGF+Ig) at our standard injection concentration (10 ng/ul), suggesting that the Ig domain may play a role in restricting EGF signaling in certain contexts. We therefore injected our LIN-3 (OE) constructs at a lower concentration (0.8 ng/ul), and we obtained several lines of each. We found, contrary to our expectation, that the SISS-1 IgD does not reduce vulval induction by the LIN-3 EGF domain ( Fig. 1C ). Together these findings suggest that the SISS-1 IgD does not function to suppress EGF signaling within the vulval epithelium. We wished to examine vulval induction in one more context, driving SISS-1 expression specifically in the anchor cell (AC). We placed our secreted EGF variants under an AC-specific promoter (Hwang & Sternberg 2004) in a *lin-3 ( e1417 )* mutant, which is vulvaless (Horvitz & Sulston 1980) due to lack of *lin-3* expression in the anchor cell (Hwang & Sternberg 2004). We found that, as with widespread expression, anchor cell-specific expression of the secreted SISS-1 EGF domain has no detectable vulval induction activity regardless of whether the Ig domain is present, while sec- LIN-3 EGF restores vulval induction to the *lin-3 ( e1417 )* mutant ( Fig. 1D ). Our findings indicate that the inability of SISS-1 to promote vulval induction is not attributable to its IgD.

We next examined the impact of the SISS-1 IgD in EGF(OE) sleep. We found the IgD to be critical for the hypersomnia produced by SISS-1 (OE) ( Fig. 1E ), consistent with the requirement for the SISS-1 IgD in endogenous sleep (Jones & Van Buskirk 2025). This IgD-dependence is observed with both pro- SISS-1 and sec- SISS-1 ( Fig. 1E ) and thus is not attributable to an impact on shedding. We wished to examine whether the SISS-1 IgD impacts LIN-3 (OE) sleep, but under our standard induction conditions our LIN-3 EGF low-concentration transgenes are not sleep-promoting ( Fig. 1E ). We therefore used a stronger induction condition, and we found that addition of the SISS-1 IgD to LIN-3 mildly reduces its sleep-promoting activity ( Fig. 1F ), counter to our expectation.

Together our data argue against a model in which the SISS-1 IgD functions to potentiate EGF signaling specifically within the nervous system, and it remains unclear why SISS-1 (OE) can promote sleep but not vulval induction. One model to explain this discrepancy is that SISS-1 is a low-affinity EGFR ligand relative to LIN-3 , and that vulval induction involves a higher signaling threshold. However, this is unlikely to be the whole story, given that under mild overexpression conditions (33˚C transgene induction), low levels of LIN-3 can trigger vulval induction but not sleep ( Fig. 1C, E). Given that sleep and vulval induction appear to rely on different effectors of EGFR activation (Van Buskirk & Sternberg 2007; Moghal & Sternberg 2003), another possibility is that both ligands can interact with LET-23 /EGFR within VPCs and within sleep-promoting neurons, but only LIN-3 triggers the appropriate downstream signaling in both cell types. Last, the reason for the strict IgD-dependence of the *C. elegans* Ig-EGFs is unclear and is currently under investigation.

## Methods

**Transgene construction:** All pro- *siss-1* fragments were based on *siss-1 b* (F28E10.2b) (Sternberg et al. 2024), as previously described (Hill et al. 2024). Gene fragments were synthesized by Twist Biosciences. Pro- *siss-1* and sec- *siss-1* gene fragments were synthesized as previously described (Hill et al. 2024) but with the IgD (Pro11-Arg109) deleted, corresponding to nucleotides 31-327 of the *siss-1 b* transcript. The sec- *lin-3* EGF gene fragment contains nucleotides 423-615 (Ala141-Ile205) of the *lin-3 c* coding sequence (F36H1.4.c), with an added 5' synthetic signal peptide (Perry et al. 1993) and a 3' STOP codon (TAA). Sec- *lin-3* EGF+Ig contains the *siss-1* IgD (Pro11-Arg109) between the signal peptide and the *lin-3* coding DNA. EcoRV and KpnI were used to shuttle gene fragments into appropriate vectors. pPD49.83 (Addgene plasmid #1448) contains the heat-inducible *hsp-16.41* promoter and was used to produce pJJ1, 2, 5, and 7. pCV45 (Van Buskirk, unpublished) contains an anchor cell enhancer element (Hwang & Sternberg 2004) and a Δ *pes-10* minimal promoter (Seydoux & Fire 1994), and was used to produce pJJ3, 4, and 6. For transgenes without detectable EGF activity in our assays, the corresponding plasmids (pJJ1, pJJ2 and pJJ4) were sequenced (Laragen Inc.) from the promoter through the 3'UTR.

**Representative lines** : Multiple independent transgenic lines were obtained for each construct. In most cases the independent lines behaved similarly and either one line was chosen as representative, or data from multiple lines was pooled. For the low-concentration sec- LIN-3 EGF(low) and sec- LIN-3 EGF+Ig(low), we chose the line from each that showed the highest VPC induction activity and used these for all assays.

**Growth conditions:** Animals were cultured on 60x15 mm (15 ml) nematode growth media (NGM) plates seeded with OP50 *E. coli* . For sleep and vulval induction experiments involving heat-inducible expression constructs, animals were grown at approximately 16°C prior to heat shock and approximately 20°C following heat shock. For VPC induction experiments using anchor cell expression constructs, animals were grown anywhere between approximately 16°C and room temperature.

**Sleep experiments:** About 30-50 well-fed young adult animals were transferred to 35x10 mm (5 ml) NGM plates seeded with OP50 *E. coli.* Plates were sealed with Parafilm and placed lid-side-up in a circulating water bath at either 33°C for 20 min (standard heat shock) or 35°C for 25 min (intense heat shock). Following heat shock, plates were immediately placed on ice for 1 min to bring them back to room temperature. Plate lids were then removed for the duration of the assay. Plates were gently moved to the center of microscope stage and left undisturbed for 30-60 sec before scoring. Sleep was defined as a lack of movement and pharyngeal pumping over a 3-second observation, and typically the first 25 animals in view with mouth on the bacterial lawn were scored. The experimenter was blind to genotype except in the case of hs: LIN-3 EGF (high) animals, which displayed a plate-level Multivulva phenotype, likely due to leaky transgene expression.

**Vulval Induction Scoring** : Vulval development was scored during the mid-L4 stage on a Zeiss Axio Imager A2. Animals were mounted on thin agarose pads in 1 mM levamisole in M9. The experimenter typically scored two or more genotypes at a time, blind. For scoring vulval induction by ACEL:EGF constructs in a *lin-3 ( e1417 )* background, animals with an invagination of the vulval epithelium at the normal location but of reduced size relative to wild type were scored as hypo-induced. Animals displaying an abnormally large invagination, or an extra invagination alongside a wild-type vulva, or more than two invaginations of any size were scored as hyper-induced. For scoring heat-inducible EGF(OE) in a wild-type background, mid-to-late L2 animals on 60x15 mm (15 ml) plates were incubated in a 33°C water bath for 30 min and returned to 16°C until reaching the L4 stage. The number of VPCs induced was inferred by the number and size of invaginations of the vulval epithelium as described previously (Mendez et al. 2025).

**Statistics:** Statistical analyses except for Holm correction were run in GraphPad Prism 10, www.graphpad.com. Holm correction was run in R 4.5.2 (R Core Team, 2025) using *p* values obtained from Prism's Fisher's exact test, and ChatGPT (OpenAI, 2025) was consulted to generate R code. Holm correction R script for Figure 1C and Figure 1D can be found at https://github.com/Jesse-G-Jones/PublicationScripts/blob/main/fig_c_holm_correction.R and https://github.com/Jesse-G-Jones/PublicationScripts/blob/main/fig_d_holm_correction.R , respectively.

**Visualization** : Schematics were created using BioRender. Figure 1A can be accessed at https://BioRender.com/u9dcg8p and Figure 1B at https://BioRender.com/49ho6ht . Figure panels were arranged in GraphPad Prism.

## Reagents

**Table:** 

**Strain**	**Description**
N2	Wild isolate
CVB56	*csnEx1* [ *hsp-16.41* p::pro- *siss-1* (pCV43), *myo-2* p::GFP]
CVB57	*csnEx2* [ *hsp-16.41* p::sec- *siss-1* (pCV44), *myo-2* p::dsRED]
CVB62	*csnEx1 7* [ *hsp-16.41* p::sec- *siss-1* –Ig (pJJ2), *unc-122* p::GFP]
CVB65	*csnEx1 8* [ *hsp-16.41* p::sec- *lin-3* EGF (pJJ5), *col-12* p::dsRED]
CVB71	*lin-3 ( e1417 )* ; *csnEx1 9* [ACp::sec- *lin-3* EGF (pJJ6), *unc-122* p::GFP]
CVB74	*lin-3 ( e1417 )* ; *csnEx2 0* [ACp::sec- *siss-1* +Ig (pJJ3), *myo-2* p::GFP]
CVB77	*lin-3 ( e1417 )* ; *csnEx2 1* [ACp::sec- *siss-1* –Ig (pJJ4), *myo-2* p::dsRED]
CVB97	*csnEx8* [ *hsp-16.41* p::pro- *siss-1* –Ig (pJJ1), *myo-3* p::GFP]
CVB98	*csnEx2 2* [ *hsp-16.41* p::sec- *lin-3* EGF+Ig(low) (pJJ7), *myo-2* p::GFP, *unc-54* p::GFP, *unc-122* p::GFP]
CVB109	*csnEx2 3* [ *hsp-16.41* p::sec- *lin-3* EGF(low) (pJJ5), *col-12* p::dsRED, *myo-2* p::GFP]

N2 is available from the Caenorhabditis Genetics Center; all other strains and plasmids are available from the Van Buskirk lab upon request.
